# Oligodendrocyte precursor cells transplantation protects blood–brain barrier in a mouse model of brain ischemia via Wnt/β-catenin signaling

**DOI:** 10.1038/s41419-019-2206-9

**Published:** 2020-01-06

**Authors:** Liping Wang, Jieli Geng, Meijie Qu, Fang Yuan, Yuyang Wang, Jiaji Pan, Yongfang Li, Yuanyuan Ma, Panting Zhou, Zhijun Zhang, Guo-Yuan Yang

**Affiliations:** 1 Department of Neurology, Ruijin Hospital, School of Medicine, Shanghai Jiao Tong University, Shanghai, 200025 China; 2 Med-X Research Institute and School of Biomedical Engineering, Shanghai Jiao Tong University, Shanghai, 200030 China; 3 Department of Neurology, Renji Hospital, School of Medicine, Shanghai Jiao Tong University, Shanghai, 200025 China

**Keywords:** Stroke, Translational research

## Abstract

Blood–brain barrier damage is a critical pathological feature of ischemic stroke. Oligodendrocyte precursor cells are involved in maintaining blood–brain barrier integrity during the development. However, whether oligodendrocyte precursor cell could sustain blood–brain barrier permeability during ischemic brain injury is unknown. Here, we investigate whether oligodendrocyte precursor cell transplantation protects blood–brain barrier integrity and promotes ischemic stroke recovery*.* Adult male ICR mice (*n* = 68) underwent 90 min transient middle cerebral artery occlusion. After ischemic assault, these mice received stereotactic injection of oligodendrocyte precursor cells (6 × 10^5^). Oligodendrocyte precursor cells transplantation alleviated edema and infarct volume, and promoted neurological recovery after ischemic stroke. Oligodendrocyte precursor cells reduced blood–brain barrier leakage via increasing claudin-5, occludin and β-catenin expression. Administration of β-catenin inhibitor blocked the beneficial effects of oligodendrocyte precursor cells. Wnt7a protein treatment increased β-catenin and claudin-5 expression in endothelial cells after oxygen–glucose deprivation, which was similar to the results of the conditioned medium treatment of oligodendrocyte precursor cells on endothelial cells. We demonstrated that oligodendrocyte precursor cells transplantation protected blood–brain barrier in the acute phase of ischemic stroke via activating Wnt/β-catenin pathway. Our results indicated that oligodendrocyte precursor cells transplantation was a novel approach to the ischemic stroke therapy.

## Introduction

Blood–brain barrier (BBB) disruption is a critical pathological feature in the acute phase of cerebral disorder^1,2^. BBB consists of brain endothelial cells with their tight junctions, the basement membrane, pericytes, and astrocyte end-feet, which shields the brain against toxins and pathogens, and allows delivery of nutrients to the brain^3,4^. Brain endothelial cells are the core element of BBB^5^. Tight junction proteins of brain endothelial cells determine the BBB permeability^2^. Brain endothelial cells express high level of tight junction proteins including claudins, occludin, and zonula occludens family^6,7^. Claudin-5, the most abundant claudin of BBB, disappears in endothelial cells from 1 day to 7 days after ischemic stroke and reappears in newly repaired brain endothelial cells, which makes itself as a promising target to protect BBB injury^2^. Studies reported Wnt/β-catenin pathway played a central role in the tight junction protein formation^1,3,6^. Activation of Wnt/β-catenin pathway could upregulate tight junction proteins expression especially claudin-5.

Stem cell transplantation showed a promising potential for ischemic stroke therapy^8–11^. Oligodendrocyte precursor cells (OPCs) derived from the ventricular zone in the embryo and migrated widely through the central nervous system, comprising about 5% of cells in the adult brain^12,13^. OPCs transplantation could enhance spatial learning and memory after hypoxic ischemic injury in premature rat brain^14^. Researches implied OPCs could maintain the BBB integrity and had close interaction with brain endothelial cells during development^15^. OPCs migrated along the vasculature via CXCl12-Wnt-CXCR4 signaling^13^. OPC HIF1/2α activity could induce paracrine Wnt7 to promote Wnt-dependent angiogenesis^16^. Meanwhile OPCs were a source of Wnt7, which served as the dominant ligands acting on frizzled receptors (Fzd) on brain endothelial cells and then activated β-catenin^17^. This evidence intrigued us to hypothesize that OPCs transplantation could attenuated tight junction disruption in brain endothelial cells after cerebral ischemia.

In this study, we aim to explore: (1) whether OPCs transplantation promotes the brain function recovery in ischemic mice; (2) whether OPCs transplantation attenuates BBB breakdown and reduces brain edema at the acute phase of cerebral ischemia; and (3) whether Wnt/β-catenin pathway is involved in the effect of OPCs on endothelial cells.

## Results

### OPCs identification, transplantation, and differentiation

**Fig. 1:**
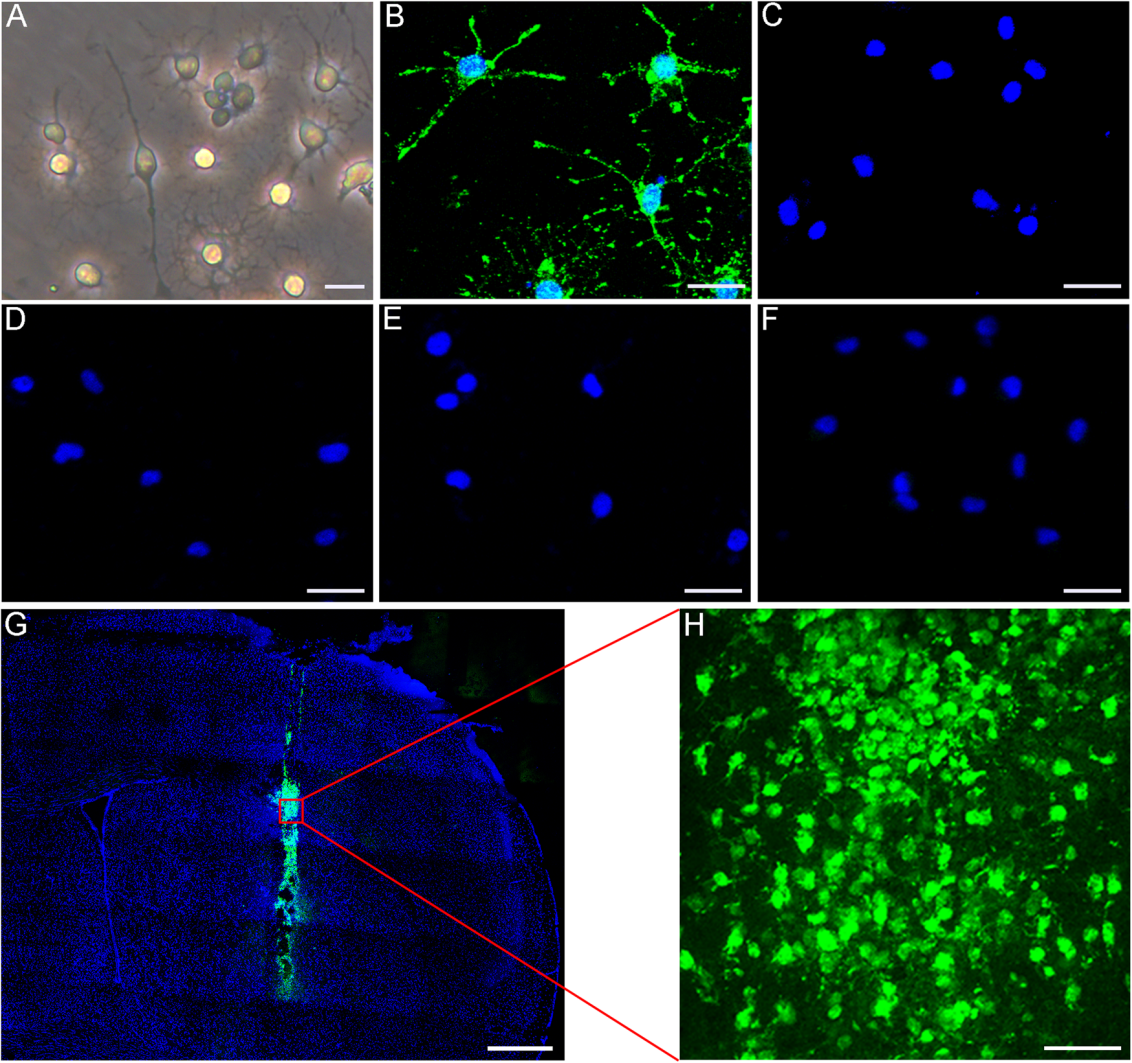
OPC identification and transplantation. **a** Cultured cells under phase-contrast microscopy. Immunofluorescence staining depicted that cultured cells were positive for NG2 (**b**), and negative for MBP (**c**), GFAP (**d**), NeuN (**e**), and Iba-1 (**f**). **g** Green fluorescent OPCs (CFDA-SE stained) were located in the ischemic hemisphere after 3 days of injection. **h** Survival of OPCs after injection. Scale bar = 10 µm (**a**), 25 µm (**b**–**f**), 500 µm (**g**), and 50 µm (**h**).

Cultured OPCs showed a bipolar or multipolar morphology under phase-contrast microscope (Fig. 1a). Immunofluorescent staining showed that the percentage of NG2^+^ cells was 93.82% (Fig. 1b and Supplementary Fig. 1E). Very few cells expressed GFAP, Iba-1, MBP or NeuN (Fig. 1c–f, the positive controls in Supplementary Fig. 1A–D). For in vivo cell tracking, we labeled OPCs with CFDA-SE. The results demonstrated that a considerable number of transplanted OPCs could survive after 3 days following middle cerebral artery occlusion (MCAO) (Fig. 1g, h).

### OPCs transplantation attenuated infarct volume and brain edema after MCAO

**Fig. 2:**
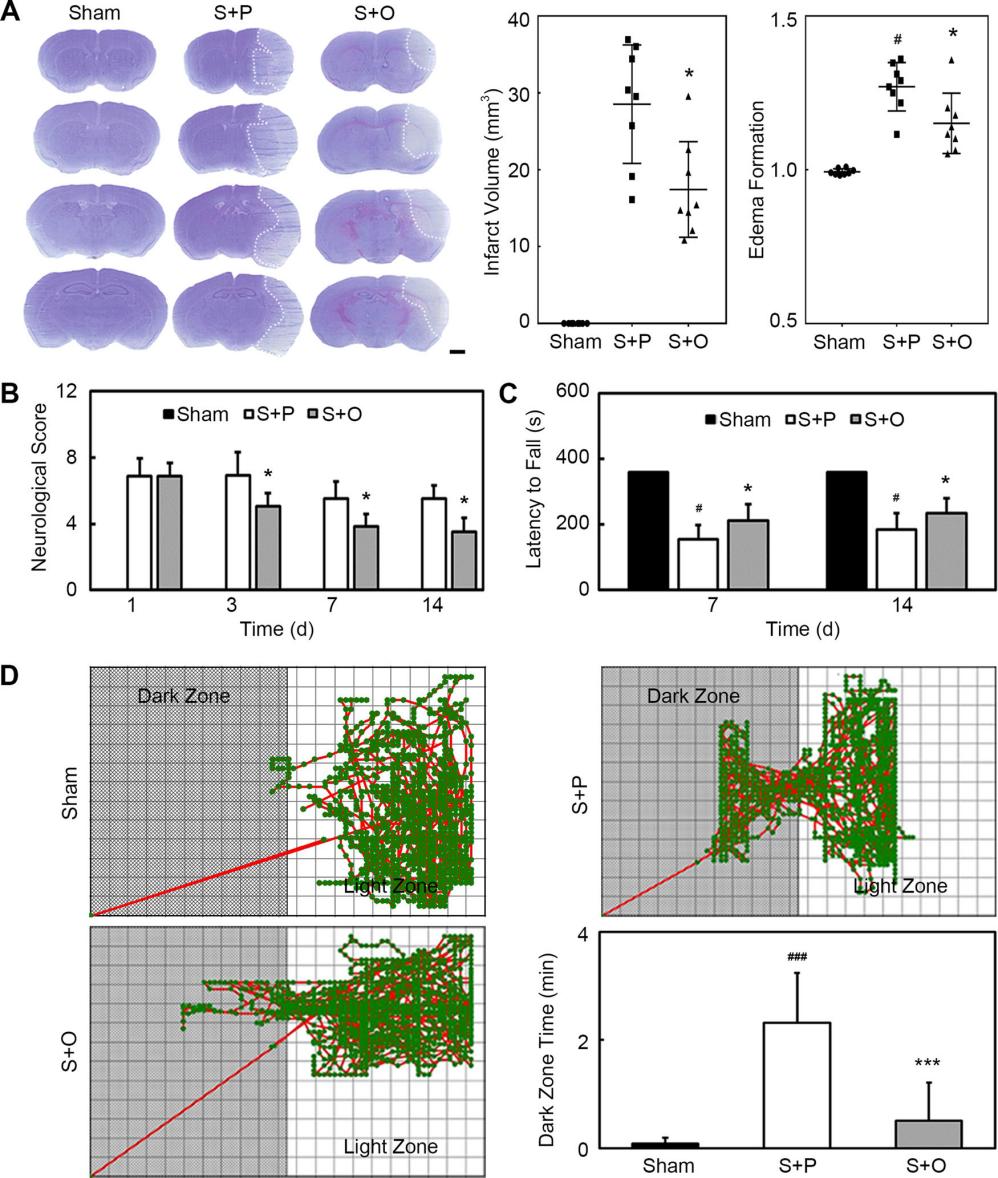
OPCs transplantation attenuated infarct volume and brain edema and improved neurological outcomes after MCAO. **a** Cresyl violet staining showed the infarction after MCAO in Sham, Stroke + PBS and Stroke + OPC groups. Bar graphs indicated that OPCs transplantation reduced infarct volume and edema formation. Scale bar = 1 mm. **b** Bar graph of neurological scores. **c** Bar graph of latency to fall. **d** Trace pictures and the bar graph of step through test showed that OPCs ameliorated the cognitive damage caused by ischemic stroke. Data are mean ± SD, *n* = 8–10 per group. *p < 0.05, ***p < 0.001, as compared with Stroke + PBS group. ^#^*p* < 0.05, ^###^*p* < 0.001, as compared with Sham group. S + P: Stroke + PBS; S + O: Stroke + OPC.

The brain edema and infarct volume were evaluated using cresyl violet staining. Results showed that the edema formation was significantly less in the OPC-treated mice compared with the phosphate buffered saline (PBS)-treated mice at 3 days after MCAO (Fig. 2a, *n* = 8–10 per group, *p* < 0.05). Infarct volume was also significantly decreased in the Stroke + OPC group compared with the Stroke + PBS group at 3 days after MCAO (Fig. 2a, *p* < 0.05).

### OPCs transplantation improved neurobehavioral recovery after MCAO

We used modified neurological severity score (mNSS) and rotarod test to evaluate the neurological function. OPCs transplantation significantly decreased neurological scores at 3, 7, and 14 days after MCAO (Fig. 2b, *n* = 8–10 per group, *p* < 0.05). Rotarod tests showed that the time staying on the rotarod was prolonged in the Stroke + OPC group compared with the Stroke + PBS group at 7 and 14 days after MCAO (Fig. 2c, *n* = 8–10 per group, *p* < 0.05). Results demonstrated that neurobehavioral deficiency was attenuated in the Stroke + OPC group compared with the Stroke + PBS group. Cognitive defect was detected by step through test at 12 days after MCAO. Step through test showed that transplanted OPCs ameliorated the cognitive damage caused by ischemic stroke (Fig. 2d, *n* = 8–10 per group, *p* < 0.001).

### OPCs transplantation sustained BBB integrity after MCAO

**Fig. 3:**
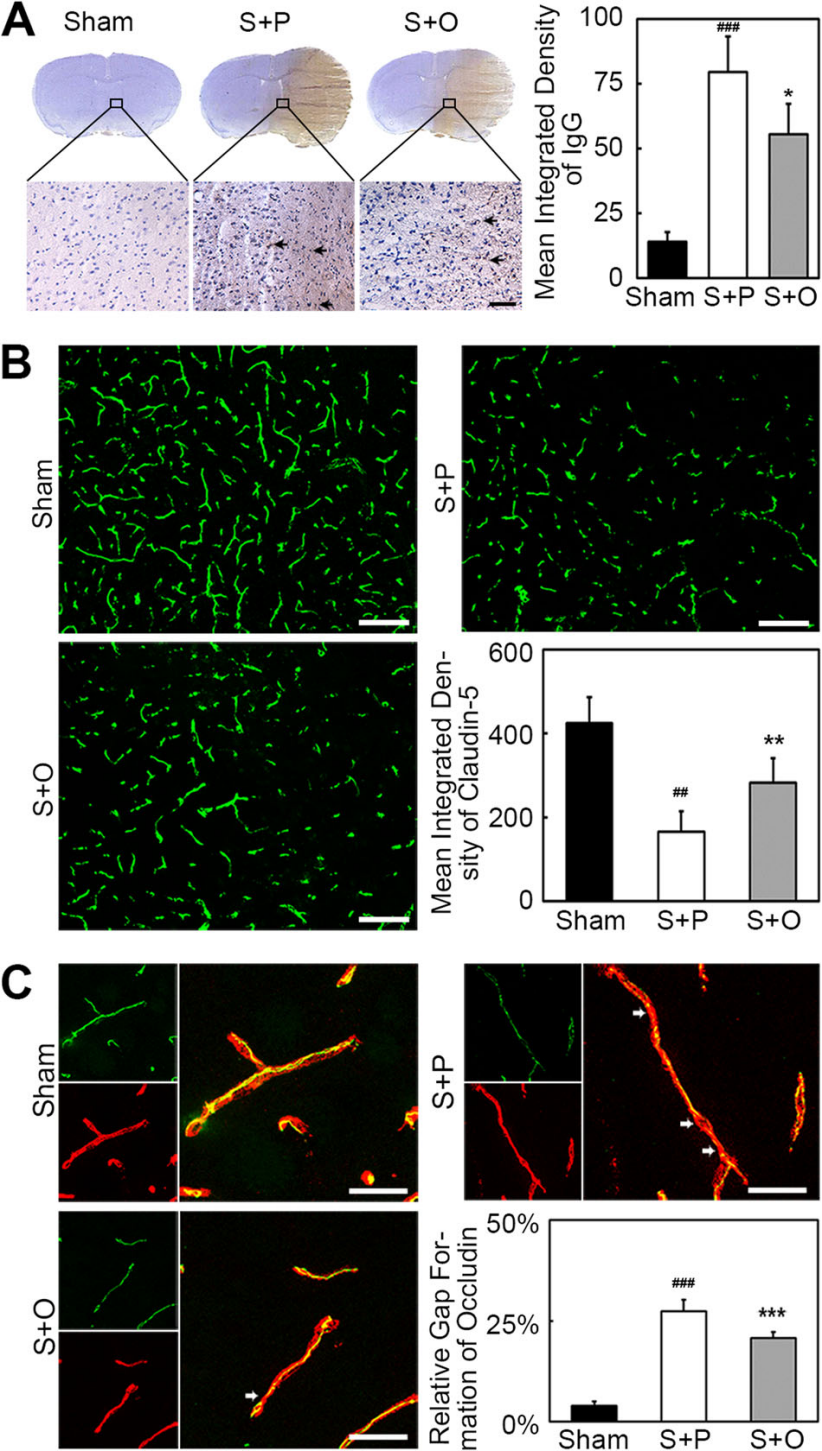
OPCs transplantation maintained the integrity of the BBB after MCAO. **a** IgG staining showed that IgG protein leaked into brain tissue in Sham, Stroke + PBS and Stroke + OPC groups. Bar graph was the quantification of leaked IgG protein, indicating less IgG protein leaked into brain tissue in Stroke + OPC group compared with Stroke + PBS group. Scale bar = 50 µm. **b** Representative images of claudin-5 (green) and endothelial marker CD31 (red) in the peri-infarct area after 3 days following MCAO and images of only claudin-5 (green) in three groups. The blank square indicated the location where the images were captured. Bar graph showed the quantification of claudin-5. OPCs alleviated the disruption of claudin-5. Scale bar = 100 µm. **c** Three-dimension reconstruction confocal microscopy images of occludin (green) and endothelial marker CD31 (red) after 3 days following MCAO in three groups. Bar graph showed the quantification of gap formation of occludin. OPCs reversed gap formation of occludin. Scale bar = 25 µm. Data are mean ± SD, *n* = 6–8 per group. *p < 0.05, **p < 0.01, ***p < 0.001, as compared with Stroke + PBS group. ^##^*p* < 0.01, ^###^*p* < 0.001, as compared with Sham group. S + P: Stroke + PBS; S + O: Stroke + OPC.

To evaluate BBB permeability after ischemic brain injury, IgG protein extravasation were measured. IgG staining showed that IgG protein leaked into brain tissue after ischemia. The quantification result showed that leaked IgG protein decreased in the Stroke + OPC group compared with the Stroke + PBS group at 3 days after MCAO (Fig. 3a, *p* < 0.05), which indicated that BBB integrity was protected through the OPC transplantation.

To investigate the mechanism of BBB disruption, we analyzed the expression of claudin-5 and occludin in cerebral vascular structures using claudin-5 and occludin/CD31 double staining. Results showed that OPCs alleviated the disruption of claudin-5 (Fig. 3b, *p* < 0.01). Occludin expression presented gap on the endothelial cell margin of cerebral microvessel after ischemic injury. Our results suggested that OPCs greatly reduced gap formation after MCAO (Fig. 3c, *p* < 0.001).

### OPCs transplantation enhanced the β-catenin expression in endothelial cells

**Fig. 4:**
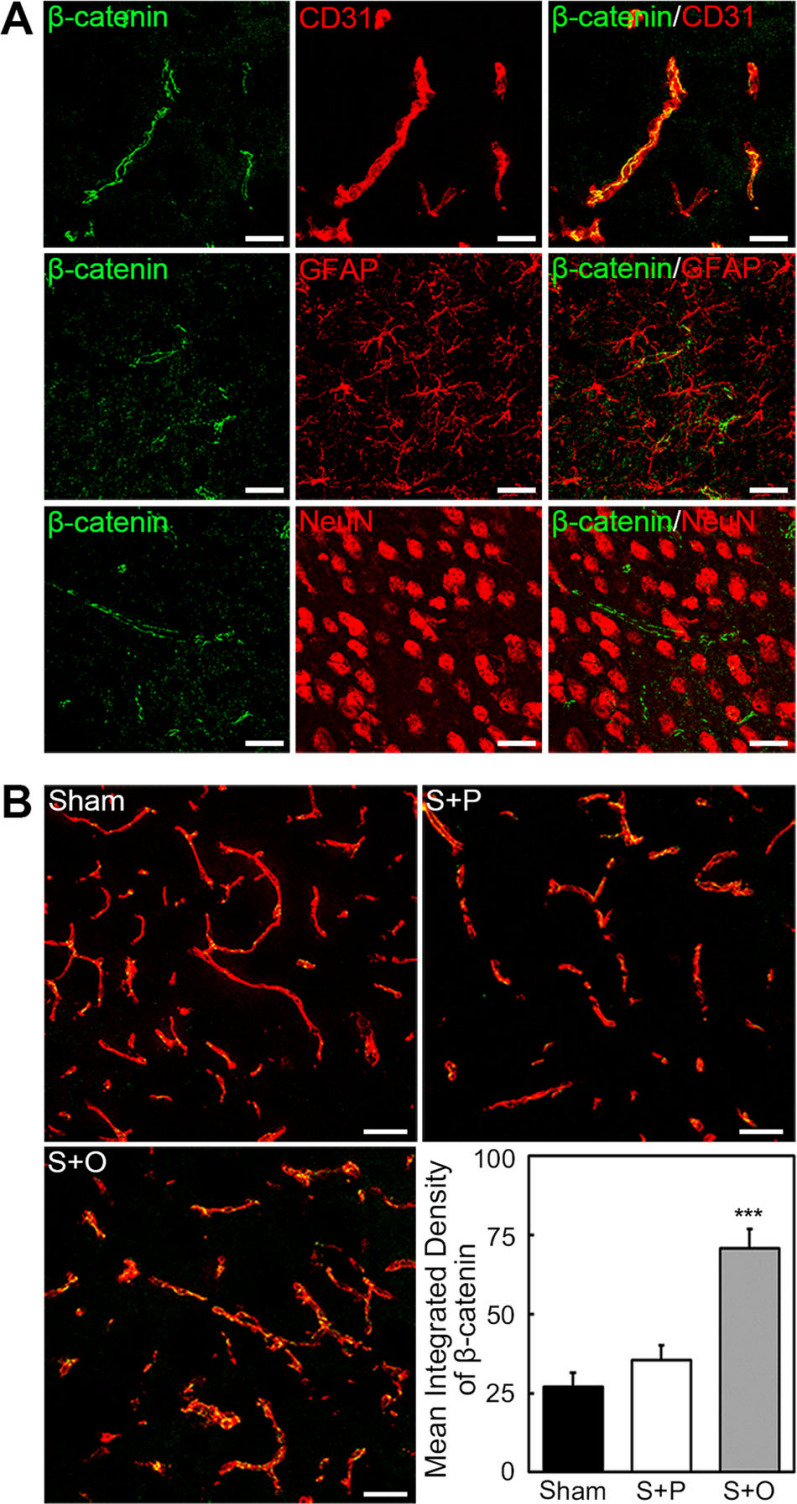
OPCs transplantation enhanced the expression of β-catenin in endothelial cells. **a** β-catenin (green) was mainly expressed on the cerebrovasculature (red). Astrocytes (GFAP^+^) and neuron (NeuN^+^) expressed little β-catenin. Scale bar = 25 µm. **b** Images of β-catenin (green) and endothelial marker CD31 (red) in the peri-infarct area after 3 days following MCAO in Sham, Stroke + PBS, and Stroke + OPC groups. Bar graph showed OPCs upregulated the expression of β-catenin. Data are mean ± SD, *n* = 6–8 per group, ***p < 0.001, as compared with Stroke + PBS group. Scale bar = 50 µm. S + P: Stroke + PBS; S + O: Stroke + OPC.

To examine whether OPCs had beneficial effect on endothelial cells after MCAO, we performed β-catenin/CD31 double staining. β-catenin was mainly expressed on the cerebral vasculature (Fig. 4a). The quantification result indicated OPCs transplantation upregulated the expression of β-catenin (Fig. 4b, *p* < 0.001).

### Inhibition of β-catenin aggravated claudin-5 disruption, BBB leakage, and neurological outcomes

**Fig. 5:**
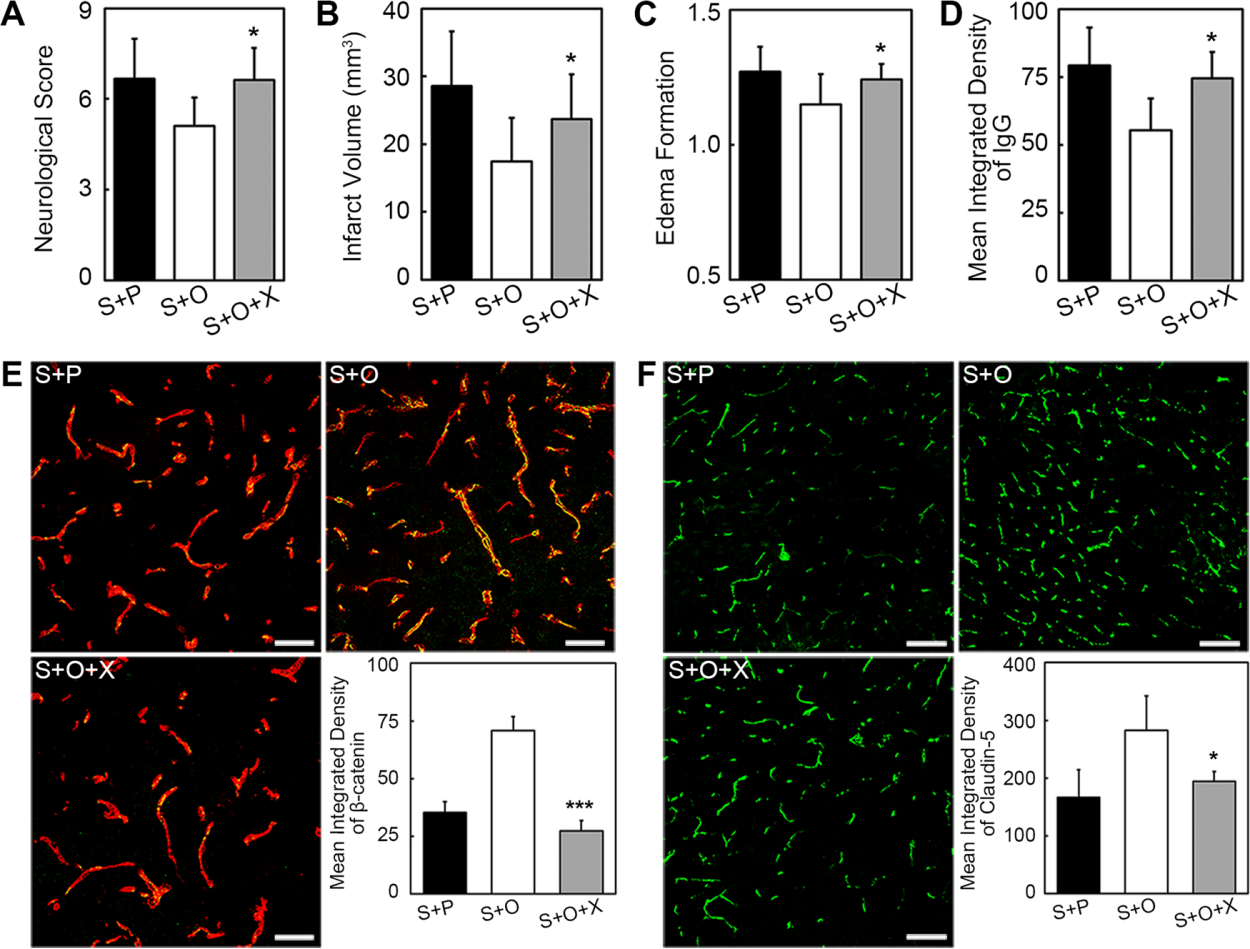
Inhibition of β-catenin aggravated the neurological outcomes, infarct volume, edema formation, and BBB disruption. Bar graphs indicated the inhibition of β-catenin hindered the beneficial effects of OPCs in neurological scores (**a**), infarct volume (**b**), and edema formation (**c**), respectively. **d** IgG staining showed the IgG leakage in Stroke + PBS, Stroke + OPC, and Stroke + OPC + XAV-939 groups after 3 days following MCAO. **e** Images showed the β-catenin (green)/CD31(red) immunofluorescence staining in above three groups. XAV-939 decreased the expression of β-catenin in endothelial cells. **f** Immunofluorescent images of claudin-5 (green) and bar graph displayed XAV-939 reversed the claudin-5 upregulation caused by OPCs. Data are mean ± SD, *n* = 6–8 per group, *p < 0.05, ***p < 0.001, as compared with Stroke + OPC group. Scale bar = 50 µm (**e**) and 100 µm (**f**). S + P: Stroke + PBS; S + O: Stroke + OPC; S + O + X: Stroke + OPC + XAV-939.

To further examine whether β-catenin was involved in the beneficial role of OPCs after ischemic stroke, β-catenin inhibitor XAV-939 was injected in OPC-treated ischemic mice for consecutive 3 days. Axin2 can degrade β-catenin and XAV-939 can stabilize Axin2 to promote β-catenin degradation^16^. We found that XAV-939 could block the beneficial effect of OPCs on neurological deficiency, infarct volume, and edema at 3 days after MCAO (Fig. 5a–c, *p* < 0.05). XAV-939 treatment reversed the protective role of OPCs on the integrity of BBB (Fig. 5d, *p* < 0.05). IgG leakage was increased after XAV-939 administration. Immunostaining images showed β-catenin level decreased in the endothelial cells of OPC-treated with XAV-939 mice (Fig. 5e, *p* < 0.001). The expression of claudin-5 was also decreased after XAV-939 administration (Fig. 5f, *p* < 0.05).

### Wnt7a secreted by OPCs activated β-catenin and upregulated claudin-5 in bEnd.3 cells after oxygen–glucose deprivation (OGD)

**Fig. 6:**
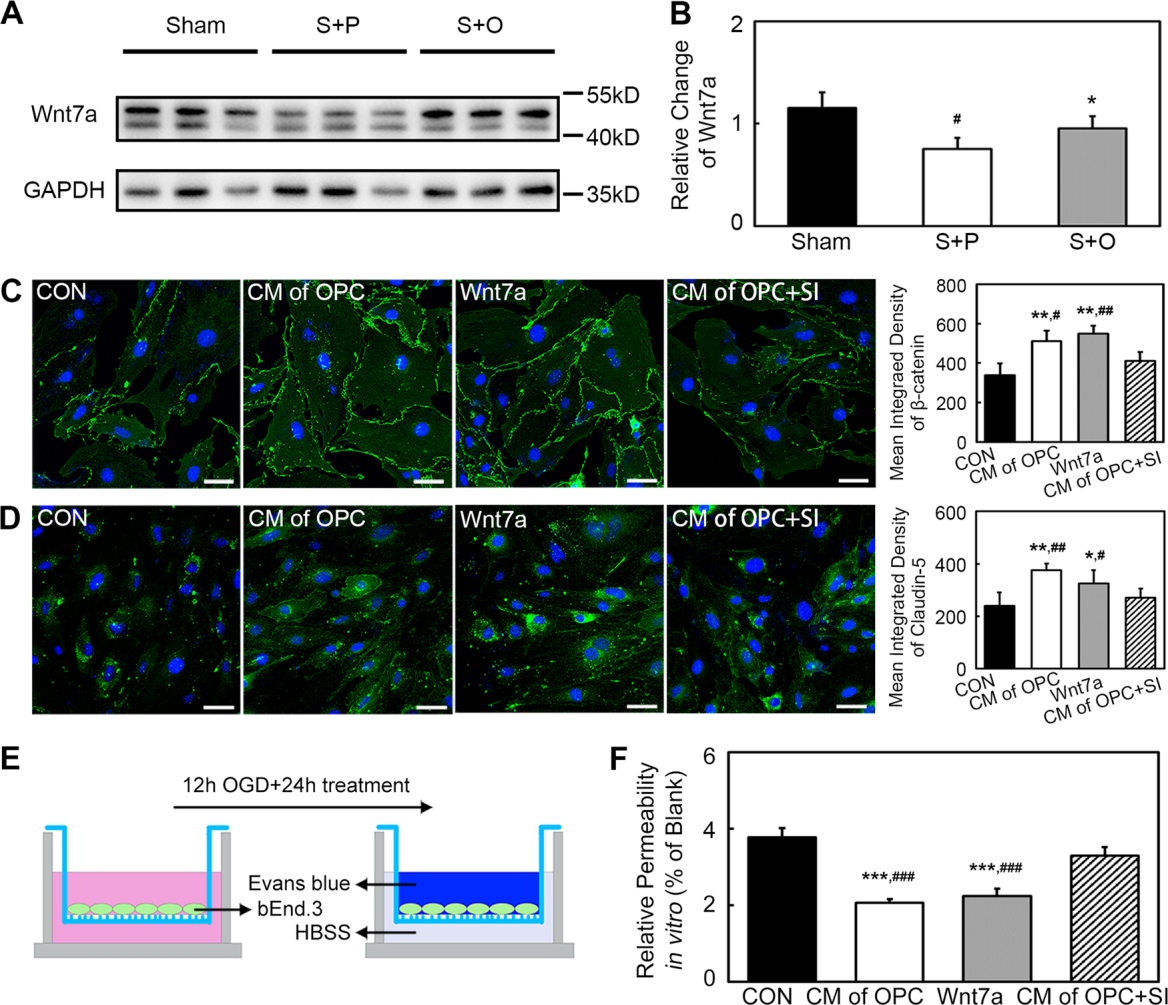
Wnt7a secreted by OPCs activated β-catenin and upregulated claudin-5 in bEnd.3 cells after OGD. **a** Western blot of Wnt7a in Sham, Stroke + PBS, and Stroke + OPC groups. **b** Western blot revealed Wnt7a increased in Stroke + OPC group after ischemia compared with Stroke + PBS group. Data are mean ± SD, *n* = 3 per group, *p < 0.05, as compared with Stroke + PBS group, ^#^*p* < 0.05, as compared with Sham group. S + P: Stroke + PBS; S + O: Stroke + OPC. **c** Images showed the β-catenin (green) expression of bEnd.3 cells in control (CON), CM of OPC, Wnt7a, and CM of OPC + SI groups at 24 h after OGD. Statistical analysis indicated CM of OPC and Wnt7a could promote β-catenin expression in bEnd.3 cells compared with control. Data are mean ± SD, *n* = 4 per group, **p < 0.01, as compared with CON group, ^#^*p* < 0.05, ^##^*p* < 0.01, as compared with CM of OPC + SI group. **d** Images showed the claudin-5 (green) expression of bEnd.3 cells in CON, CM of OPC, Wnt7a, and CM of OPC + SI groups at 24 h after OGD. Data are mean ± SD, *n* = 4 per group, *p < 0.05, **p < 0.01, as compared with CON group, ^#^*p* < 0.05, ^##^*p* < 0.01, as compared with CM of OPC + SI group. **e** Experimental setup for Evans blue determination in vitro. **f** Bar graph indicated the relative permeability of endothelial barrier in vitro. Data are mean ± SD, *n* = 4 per group, ***p < 0.001, as compared with CON group, ^###^*p* < 0.001, as compared with CM of OPC + SI group. Scale bar = 50 µm.

Western blot revealed Wnt7a level was increased in the Stroke + OPC group compared with the Stroke + PBS group (Fig. 6a, b, *p* < 0.05). Images analysis indicated both the conditioned medium (CM) of OPC and Wnt7a could increase β-catenin expression in bEnd.3 cells compared with the control (Fig. 6c, *p* < 0.01). Claudin-5 expression increased in CM of OPC (*p* < 0.01) and Wnt7a (*p* < 0.05) groups compared with the control group in bEnd.3 cells at 24 h after OGD (Fig. 6d). While knocking down the Wnt7a of OPC could block the effect of CM of OPC.

### CM of OPC and Wnt7a supported the endothelial barrier after OGD

To confirm whether CM of OPC and Wnt7a could support the endothelial barrier, in vitro endothelial permeability assay was performed (Fig. 6e). The leaked amount of Evans blue was detected for the evaluation of permeability. The Evans blue result showed that both CM of OPC and Wnt7a decreased the endothelial permeability compared with the control and CM of OPC with knocking down Wnt7a (Fig. 6f, *p* < 0.001).

## Discussion

Our study demonstrated that OPCs transplantation effectively protected BBB integrity and promoted functional recovery in ischemic mice. Wnt/β-catenin pathway activated by OPC treatment participated in the upregulation of tight junction proteins after ischemic stroke.

BBB disruption is a canonical feature of ischemic stroke^18^. Oxidation and inflammation exacerbate the loss of tight junction proteins such as claudin-5 and occludin in the microvasculature, and further increase BBB permeability^19,20^. Our data showed that OPCs transplantation significantly attenuated infarct volume and brain edema after ischemia. OPCs transplantation reduced IgG leakage, rescued claudin-5 disruption in ischemic stroke, which suggested that OPCs transplantation protected BBB integrity through reducing tight junction protein degradation. Meanwhile, OPCs transplantation ameliorated neurobehavioral deficiency and cognitive defects, which may be due to the alleviating of BBB damage.

OPCs migrated along the vasculature and Wnt signaling regulated OPC-endothelium interaction during CNS development^13^. Our results supported that transplanted OPCs upregulated the β-catenin expression in endothelial cells. The inhibition of β-catenin by XAV-939 blocked the beneficial effects of OPCs on neurological outcomes, infarct volume, and edema formation. Furthermore, the inhibition of β-catenin reversed the protective role of OPCs on the integrity of BBB and aggravated claudin-5 disruption. The level of claudin-5 had positive correlation with that of β-catenin. The result supported that OPCs transplantation increased the expression of claudin-5 via upregulating β-catenin in endothelial cells.

Previous study reported that OPCs could secret large amounts of Wnt7a, which served as the dominant ligands in Wnt signaling and acted on its receptor Fzd on brain endothelial cells^16,17,21^. Wnt ligands produced by immature neuroectodermal cells could bind to Fzd on brain endothelial cells and contribute to the induction of claudin-5^22,23^. We found that both CM of OPC and Wnt7a could upregulated β-catenin expression in bEnd.3 cells. Meanwhile, claudin-5 expression in bEnd.3 cells also increased in CM of OPC and Wnt7a groups. This indicated CM of OPC and Wnt7a upregulated claudin-5 via Wnt/β-catenin signaling. The beneficial effect of CM of OPC might be largely due to Wnt7a.

**Fig. 7:**
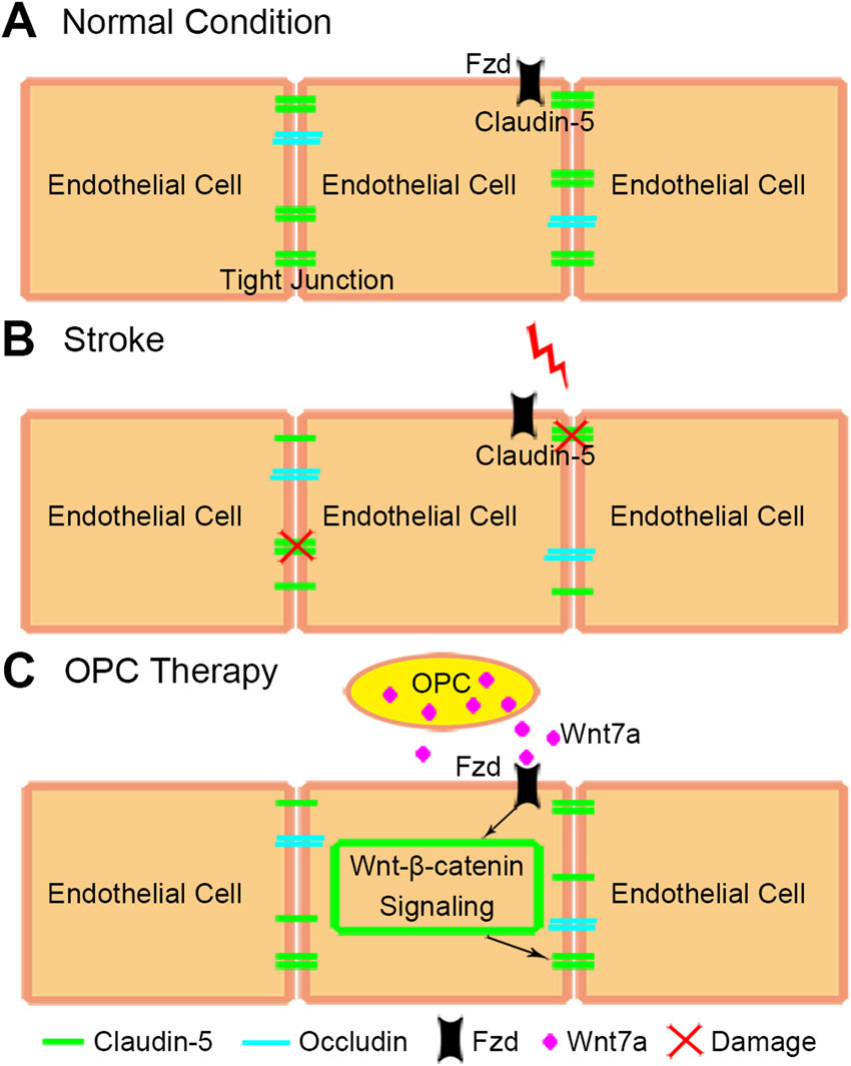
The schematic diagram showed the possible mechanism of OPC therapy for stroke. **a** In the normal condition, endothelial cells expressed tight junction proteins such as claudin-5 and occludin to keep the integrity of BBB. **b** The tight junction proteins were disrupted by oxidative stress and inflammation after stroke. **c** In the OPC therapy, transplanted OPCs released Wnt7a which acting on the corresponding receptor Fzd in the membrane of endothelial cells. Then Wnt/β-catenin pathway was activated and upregulated claudin-5.

Besides Wnt7a, OPCs can secret trophic factors, such as IGF1, GDNF, and TGF-β, providing trophic signals to neighboring cells^15,24^. These trophic factors may also be possible to regulate tight junction in brain endothelial cells. The cross talk between OPCs and brain endothelial cells regulate the BBB^15,24^. Present study supported the following possible mechanism between OPCs and brain endothelial cells: in the normal condition, endothelial cells expressed tight junction proteins such as claudin-5 and occludin, which played an important role in the integrity of BBB. In ischemic stroke, the tight junction proteins could be degraded. Transplanted OPCs released Wnt7a, which acting on the corresponding receptor Fzd in the membrane of endothelial cells, then Wnt/β-catenin pathway was activated and upregulated claudin-5 (Fig. 7).

In conclusion, we demonstrated that OPCs transplantation promoted stroke recovery in ischemic mice. OPCs protected BBB in the acute phase of cerebral ischemia via activating Wnt/β-catenin pathway of endothelial cells. OPCs transplantation is a novel approach to the ischemic stroke therapy.

## Materials and methods

### Experimental protocol

Animal procedures and protocols were approved by the Institutional Animal Care and Use Committee of Shanghai Jiao Tong University, Shanghai, China. All studies were conducted in accordance with the US National Research Council’s Guide for the Care and Use of Laboratory Animals, the US Public Health Service’s Policy on Humane Care and Use of Laboratory Animals and Guide for the Care and Use of Laboratory Animals. Animal studies were reported according to ARRIVE guidelines. Sixty-eight adult male ICR mice weighing 28–30 g (JSJ, Shanghai, China) were used in the study. Animals were fed normally in the SPF proved animal facility in Shanghai Jiao Tong University, China. Mice were randomly divided into four groups: stroke + OPC group, stroke + OPC + XAV-939 group, stroke + PBS group, and sham group, *n* = 8–16 per group.

### OPCs isolation and identification

The brain cortex was dissected from P1 Sprague-Dawley rat pups as described^25^. Brain tissue was dissociated into a single-cell suspension by trypsin at 37 °C for 10 min. After the suspension was filtered with a 70-µm filter, cells were seeded on poly-d-lysine (PDL, Sigma, St. Louis, MO) coated culture flasks in DMEM (Gibco, Carlsbad, CA) with 10% fatal calf serum (Gibco). Eight to ten days later, the microglia were separated from glia cell mixtures after 30 min of culture by a 220 rpm shake and then OPCs were collected by 20 h of culture by a 200 rpm shake. Collected cells were injected into the mouse striatum or seeded on a PDL-coated culture dish in Neurobasal-A (Gibco) containing 2% B27 (Gibco), 10 ng/ml PDGF-AA (Gibco), 10 ng/ml bFGF (Peprotech, Rocky Hill, NJ), and 2 mmol/l glutamine (Gibco).

For identification, cells were fixed with 4% paraformaldehyde and blocked by 10% bovine serum albumin. Then OPCs were incubated with primary antibodies against PDGFR-α (1:100, Santa Cruz Biotechnology, Santa Cruz, CA), NG2 (1:200, Millipore, Bedford, MA), GAFP (1:200, Millipore), MBP (1:200, Abcam, Cambridge, UK), NeuN (1:200, Millipore), and Iba-1 (1:200, WAKO, Osaka, Japan) at 4 °C overnight. Cells were incubated with the fluorescence conjugated second antibodies 37 °C for 1 h.

### Mouse model of transient MCAO

Transient MCAO was performed as described previously^26,27^. Mice were anesthetized by ketamine/xylazine (100 mg/kg, Fujian Gutian Pharmaceutical Co., Ltd, Gutian, China)/xylazine (10 mg/kg, Sigma) and placed in the supine position. After the isolation of the left common carotid artery, external carotid artery, and internal carotid artery, the origin of middle cerebral artery was occluded by a silicone-coated 6–0 suture (Covidien, Mansfield, MA). Reperfusion was performed after 90 min of MCAO by withdrawing the suture. The success of occlusion was assessed by a laser Doppler flowmetry (Moor Instruments, Devon, UK) with a decrease of cerebral blood flow at least 80% of the baseline. Sham mice were conducted the same procedure except the insertion of suture.

### OPCs transplantation and inhibition of β-catenin

Animals were randomly divided into following groups as Stroke + PBS, Stroke + OPC, and Stroke + OPC + XAV-939. OPCs or PBS (as a control) were injected at 24 h after MCAO. Before transplantation, OPCs were labeled with carboxyfluorescein diacetate-succinimidyl ester (CFDA-SE, Beyotime, Shanghai, China) for the tracking. Mice were anesthetized and received stereotaxic transplantation. A microsurgical drill made a small skull hole 2 mm lateral to the bregma. An amount of 6 × 10^5^ OPCs was suspected in 5 µl PBS and slowly injected into the left striatum at 2 mm lateral to the bregma and 3 mm under the dura (AP = 0 mm, ML = 2 mm, DV = 3 mm) at a rate of 1 µl/min by the 10 µl Hamilton syringe (Hamilton, Bonaduz, Switzerland). The same amount of PBS was injected as control. For the OPC plus XAV-939 group, XAV-939 (40 mg/kg, MCE, Monmouth Junction, NJ) was injected intraperitoneally once a day^16,28^. The mice were injected i.p. daily with cyclosporine A (5 mg/kg, Sigma) for immunosuppression after cell transplantation.

### Neurobehavioral function examination

An investigator who was blinded to the experimental treatment performed mNSS to evaluate the neurological function at 1 day, 3 days, 7 days, and 14 days after MCAO^29^. The mNSS ranged from 0 to 14 and included motor, sensory, balance, and reflex tests.

Rotarod test was performed at 7 and 14 days after MCAO. Mice were trained on a rotating rod at 20 rpm for 3 consecutive days before MCAO. Mice were placed on the rod for adaption, after which the rod was continuously accelerated to 40 rpm. The time mice stayed on the rod (latency to fall) was recorded.

Step through test was performed at 12 and 13 days after MCAO. At the first day of the test, mice were put into the light space of smart cage (AfaSci Research Laboratories, Redwood City, CA) with an electric stimulator at the base. The mouse received an electric shock once it stepped into the dark box. After 24 h, mice were put into the smart cage again without electric stimulation and monitored for 10 min. The trace was recorded by the infrared detector and analyzed by the smart cage software.

### Measurement of brain infarct volume and edema formation

Mice were sacrificed at 3 days after MCAO and brains were cut into a series of 20 µm thick coronal sections. We performed cresyl violet staining (Sigma) to measure the brain infarct volume and edema formation. Infarct volume was calculated by ImageJ software (National Institutes of Health, Bethesda, MD) as described previously^18,26^. Edema formation was calculated by the ipsilateral volume divided by contralateral volume.

### BBB permeability assay

The leakage of IgG was detected to assess BBB permeability^18,27^. Briefly, brain slices were fixed with 4% paraformaldehyde and incubated with Universal ABC Kit (Vector Labs, Burlingame, CA). After that, the immunoreactivity was visualized by DAB reagents (Vector Labs) and slices were counterstained with hematoxylin. We selected four slices from anterior to posterior coronal coordinates in each mouse for the statistical analysis. Three fields along the ischemic penumbra area for each slice were randomly collected and analyzed with ImageJ software (National Institutes of Health) for mean integrated density analysis.

### Immunohistochemistry determination

Brain slices were fixed with methanol at 4 °C for 10 min and blocked with diluted donkey serum (Jackson ImmunoResearch, West Grove, PA) for 60 min at room temperature. Slides were incubated with primary antibodies of CD31 (1:200, R&D system, Minneapolis, MN), occludin (1:200, Life Technologies, Carlsbad, CA), claudin-5 (1:200, Life Technologies), β-catenin (1:100, Abcam), GFAP (1:500, Millipore), NeuN (1:50, Millipore), and Wnt7a (1:100, Abcam) overnight at 4 °C. After rinsing three times with PBS, brain slices were incubated with the fluorescence conjugated second antibodies for 1 h at room temperature. Immunofluorescence photos were collected by a confocal microscope (Leica, Solms, Germany). For claudin-5 and β-catenin quantification, mean integrated density was measured by ImageJ software (National Institutes of Health). For occludin staining, the gap length was presented as percentage (%) of whole tight junction staining^26,27^. We randomly chose at least four vessels in the peri-focal region per brain section, and total eight sections per animal. Then we measured the length of vessel and gap by ImageJ software (National Institutes of Health). Gap length was presented as percentage (%) of gap length in whole vessel.

### Real-time PCR and western blot analysis

The real-time PCR assay and western blot were performed as described previously^26^. The two stage RT-PCR amplification parameters were 95 °C for 30 s followed by 40 cycles of 95 °C for 5 s and 60 °C for 30 s. Wnt7a mRNA expression level was normalized to reference gene GAPDH and displayed as relative expression of mRNA by 2^−Δct^ method. Wnt7a forward primer was CTC GCC ATT AGA AAA GTT CC, reverse primer was AAG CCT CTT ATC CAG TGG TT. GAPDH forward primer was GAT GGT GAA GGT CGG TGT GA, the reverse primer was TGA ACT TGC CGT GGG TAG AG.

For western blot analysis, the membranes were blocked with 5% skim milk and incubated with primary antibodies against Wnt7a (1:500, Abcam) and GAPDH (1:1000, Abcam) overnight at 4 °C. After washing with TBST buffer, the membranes were incubated with HRP-conjugated secondary antibody.

### Wnt7a siRNA interference in OPCs

Sequences of interfering siRNA segments were as follows: 5-GCCUUCACCUAUGCAAUUATT-3. Non-targeting siRNA with scramble served as a negative control (NC).

OPCs were seeded at a density of 5 × 10^5^ cells per well in six-well plates. After 24 h, the CM was collected. Twenty micromolars FAM-siRNA (8 µl, Genepharma, Shanghai, China) and 3.75 µl Lipofectamine® 3000 (Invitrogen) were each diluted by 125 µl Opti-MEM (Gibco). Diluted siRNA and Lipofectamine® 3000 were mixed and added into the 1750 µl culture medium of OPCs. After 24 h of interference, we changed new culture medium and after 48 h of interference, the CM and cell proteins of three groups (Blank, NC and siRNA) were collected.

### bEnd.3 cell culture and OGD experiment

bEnd.3 cells were purchased from the China Center for Type Culture Collection (Wuhan, China) and were grown in DMEM (Gibco) with 10% fetal bovine serum (FBS, Gibco). In OGD, bEnd.3 cells were seeded in six-well plates at a density of 2 × 10^5^ cells per well and incubated with FBS and glucose free DMEM at 5% CO_2_ and 95% N_2_ atmosphere using an airtight chamber for 12 h. Then the medium was changed with 70% normal DMEM with 30% Neurobasal-A (control), CM of OPC, CM of OPC with Wnt7a siRNA (CM of OPC + SI), or Neurobasal-A with Wnt7a protein (50 ng/mL, R&D system)^30^. After 24 h, bEnd.3 cells were performed immunostaining to test claudin-5 and β-catenin expression.

### In vitro BBB permeability assay

bEnd.3 cells were plated on PDL-coated 3-µm pore size transwell chambers of 12-well plates (Corning, New York, NY) at a density of 2 × 10^5^ cells per well. When cells reached confluency, they were treated with OGD for 12 h. Then bEnd.3 cells were treated with 70% normal DMEM with 30% Neurobasal-A (control), CM of OPC, CM of OPC + SI or Neurobasal-A with Wnt7a protein (50 ng/mL, R&D system) for 24 h.

In Evans blue determination, the upper and bottom chambers were washed with Hank’s balanced salt solution (HBSS). Evans blue staining solution was 0.25% Evans blue (Sigma) and 4% bovine serum albumin in HBSS. HBSS (1.5 ml) was added to the bottom chamber, and Evans blue staining solution (0.5 ml) was added to the upper chamber. Then the plate was incubated at 37 °C for 5 min. The solution in the bottom chamber was collected, and the amount of Evans blue was quantified at 610 nm by a spectrometer (Biotek, Winooski, VT)^19,31^. The chamber without bEnd.3 cells was as the blank group. The permeability was presented as the percentage compared with the blank group.

### Statistical analysis

Sample size was determined according to our previous publications for similar outcomes^18,26,27^. Samples size was estimated using a type I error rate of 0.05 and a power of 0.8 on a two-sided test by power analysis. Analysis were performed by SPSS. Parametric data were analyzed using one-way ANOVA followed by Tukey’s post hoc test. Data were expressed as mean ± SD. A probability value less than 0.05 was considered significant.

## Supplementary information

S1

S2

Supplementary figure legends
