# Supplementary figures and images for: Oligodendrocyte precursor cells transplantation protects blood–brain barrier in a mouse model of brain ischemia via Wnt/β-catenin signaling
Source: Cell Death Dis. 2020 Jan 6;11(1):9. doi: 10.1038/s41419-019-2206-9 (PMC6944692; doi:10.1038/s41419-019-2206-9)

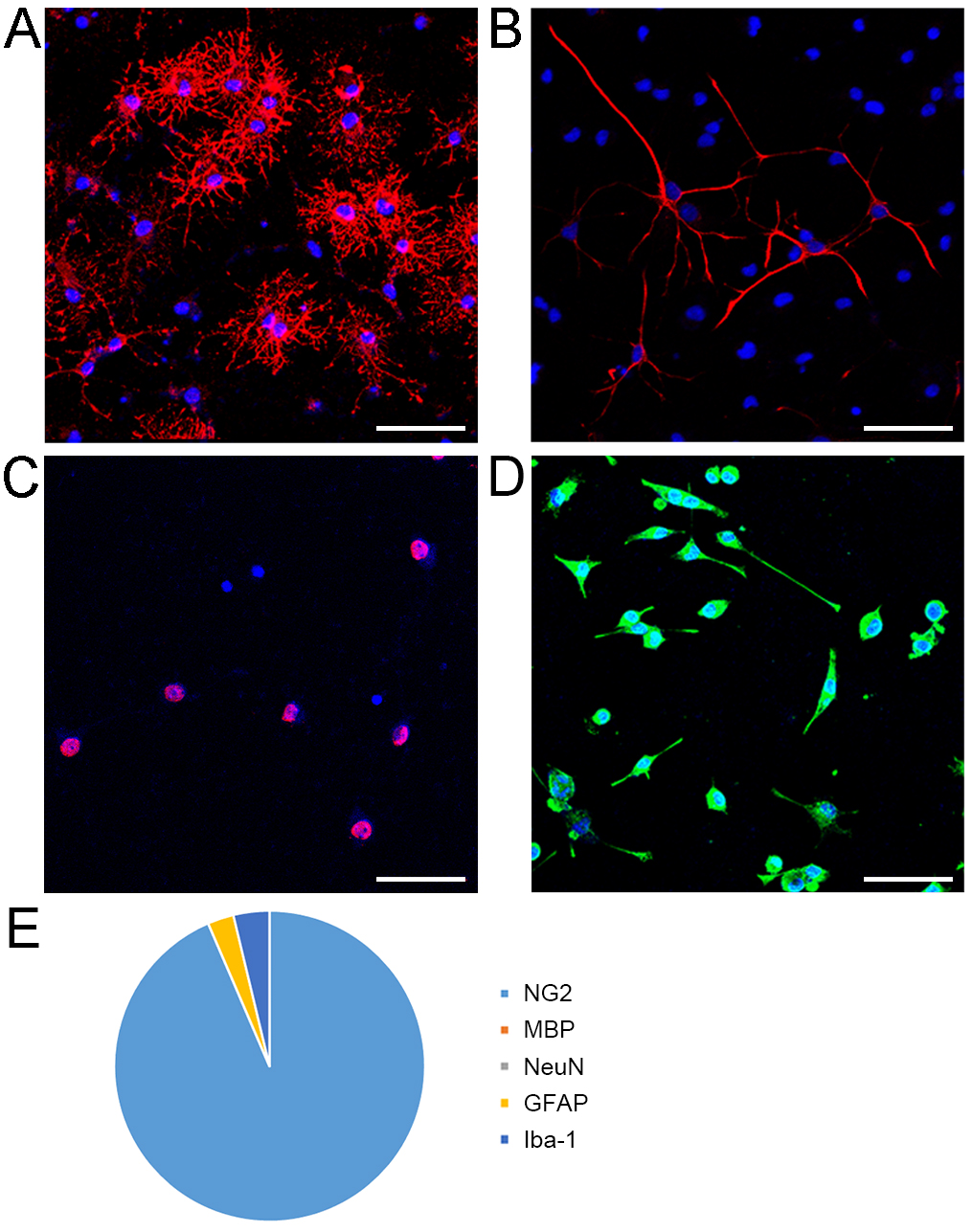

Supplement: Supplementary file 1 — S1 [file 41419_2019_2206_MOESM1_ESM.jpg]

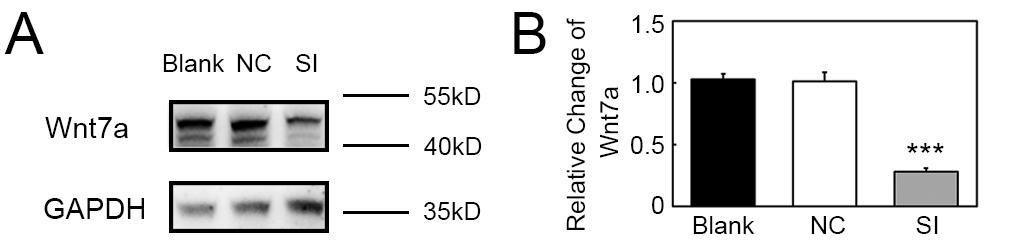

Supplement: Supplementary file 2 — S2 [file 41419_2019_2206_MOESM2_ESM.jpg]
