# Supplementary material for: Oligodendrocyte precursor cells transplantation protects blood–brain barrier in a mouse model of brain ischemia via Wnt/β-catenin signaling
Source: Cell Death Dis. 2020 Jan 6;11(1):9. doi: 10.1038/s41419-019-2206-9 (PMC6944692; doi:10.1038/s41419-019-2206-9)
Supplement: Supplementary file 3 — Supplementary figure legends [file 41419_2019_2206_MOESM3_ESM.docx]

**Suppl. Fig. 1 Positive controls for the cell staining and the quantification of NG2+ cells.**

(**A**) Positive control for the MBP staining. The MBP+ cells were primary OPCs cultured for 8 days. (**B**) Positive control for the GFAP staining. The GFAP+ cells were primary astrocytes. (**C**) Positive control for the NeuN staining. The NeuN + cells were primary neurons. (**D**) Positive control for the Iba-1 staining. The Iba-1 + cells were BV2 cells (microglia cell line). (**E**) The pie graph showed the percentages of NG2+, MBP+, NeuN+, GFAP+, and Iba-1+ cells, respectively. Scale bar=50 µm (A-D).

**Suppl. Fig. 2 Knockdown of Wnt7a in OPCs.**

(**A**) Western blot of Wnt7a expression in Blank, NC, and SI groups. Blank: without siRNA; NC: with negative control siRNA; SI: with Wnt7a siRNA. (**B**) Bar graph showed that Wnt7a siRNA knocked down the Wnt7a expression in cultured OPCs successfully. Data are mean$\pm$SD, n=3 per group, ****p*<0.001, as compared to Blank group.
